# The Measurement and Application of Critical Speed and *D*′ in Running: A Scoping Review

**DOI:** 10.1007/s40279-026-02410-x

**Published:** 2026-04-03

**Authors:** Mitchell Anderson, Surendran Sabapathy, Blayne Arnold, Clint Bellenger, Filip Kolodziej, Phillip Bellinger

**Affiliations:** 1 School of Allied Health, Sport and Social Work, Griffith University, Southport, QLD Australia; 2 Alliance for Research in Exercise, Nutrition and Activity (ARENA), Allied Health and Human Performance, University of South Australia, Adelaide, SA Australia; 3 Griffith Sport Science, Griffith University, Southport, QLD Australia

## Abstract

Critical speed (CS) represents the maximal metabolic steady state in running, and *D*′ represents the finite distance capacity above CS. However, there is currently no consensus on the optimal measurement and modelling methods to obtain CS and *D*′ estimates, how CS and *D*′ influence running performance, or how CS and *D*′ have been applied within a training environment. The aims of this scoping review were to determine the specific protocols and modelling methods that have been implemented to quantify CS and *D*′ in running; the deterministic potential of CS and *D*′ for running race performance; and the application of CS and *D*′ in individualising running training prescription. The PRISMA guidelines for scoping reviews were followed. PubMed, Scopus, Embase and SPORTDiscus were systematically searched, and studies were screened for eligibility. A total of 124 studies were included in the review, of which 23 investigated CS as the maximal metabolic steady state, 68 investigated different measurement or modelling methods on CS and *D*′, 16 examined different measurement methods on CS only, six investigated *D*′ reconstitution, 26 studies explored the relationship between CS or *D*′ and running performance, and 16 applied CS and *D*′ modelling to individualise training prescription. CS has been validated as the boundary between the heavy- and severe-intensity domains while there is a far smaller body of research investigating *D*′ reconstitution; however, variations in testing protocols may influence CS and *D*′ estimation. Of importance to coaches and athletes is that CS and *D*′ balance are particularly strong determinants of middle- and long-distance running performance (mile to marathon, and 5000 and 10,000 m, for CS and *D*′ balance, respectively), and modelling of CS and *D*′ provides an avenue to individualise training prescription.

Open Science Framework (OSF) registration: https://osf.io/rq2h3.

**Supplementary Information:**

The online version contains supplementary material available at 10.1007/s40279-026-02410-x.

## Key Points

**Table:** 

Critical speed (CS) has been verified as the maximal metabolic steady state, but there exists a level of discrepancy between other physiological thresholds (e.g., maximal lactate steady state, and the second lactate threshold). There is a wide range of testing methods and subsequent modelling used to assess CS and * D*′, the finite distance capacity above CS.
CS and * D*′ balance are strongly associated with middle- and long-distance running performance (mile to marathon for CS) and competitive race finishing position (10,000 m for CS, and 5000 m and 10,000 m for * D*′ balance).
Although CS and * D*′ modelling can assist in individualising training prescription, additional research is required to improve the accuracy of * D*′ balance modelling.

## Introduction

The critical power (CP) represents the boundary between the heavy- and severe-exercise intensity domains [1]. When exercise is performed below the CP, metabolic steady-state physiology (i.e., oxygen consumption ($\dot{V}$O_2_), blood lactate concentration) may still be achieved, but when performed above the CP, key physiological responses rise inexorably to maximal values, leading to exhaustion [1]. Although the term CP is typically used in cycling, the same concept is applied in other modes of activity such as running and swimming, but termed critical speed (CS) or critical velocity (CV). There is a finite work capacity that can be expended above CP, denoted *W*′ (or *D*′ in running and swimming). Exercising above the CP depletes *W*′, and full depletion of *W*′ coincides with exhaustion [2, 3], or the inability to sustain exercise at an intensity > CP. In contrast, exercise below the CP will replenish *W*′ if there has been prior depletion [2]. The determination of CP and *W*′ can assist across an array of contexts such as physiological benchmarking [4], race performance estimation, informing race strategy [5], and quantifying training session prescription and intermittent training demand [2]. Although the use of CP and *W*′ may be broad and can be applied across many sports [6], it is likely necessary to separate the literature into specific modalities, such as running.

Unique energetic and mechanical profiles of different sports may ultimately influence the generalisability and transferability of CP and *W*′ research between sports. For example, the time course of *W*′/*D*′ reconstitution between supra-CP/CS intervals varies between cycling and running (as indicated by different recovery time constants) [3, 7]. Additionally, CP/CS values can be influenced by cycling cadence [8] and running footwear [9], which are considerations only within their respective sports. While there is an expanse of literature focusing on CP in cycling [10, 11], a comprehensive summary of the running-based literature is lacking.

Although different tests have been used for the measurement of CS and *D*′ in running [12–14], an encompassing overview on the convergent validity and agreement of the different methods has not been established. Moreover, methods for modelling the reconstitution of *D*′ in running demonstrate variability [7]. A scoping review may identify the contextual factors (e.g., recovery intensity and duration) that have (or have not) been investigated to guide future research. Additionally, multiple terms have been reported when discussing the finite work capacity (e.g., *D*′ [5], anaerobic running capacity [12], anaerobic distance capacity [15]), which may lead to confusion. There is also a line of evidence demonstrating that CS and *D*′ are determinants of endurance performance [5, 16], but there is no clear consensus on how these parameters relate to performance across a range of specific distance events. Furthermore, modelling CS and *D*′ can assist in individualising high-intensity interval training [3], but the validity of a CS model for intermittent running exercise in which the balance of *D*′ remaining may be calculated requires further clarification across the literature. Given the range of research gaps that require clarification, a scoping review is able to provide a foundational overview of the current evidence base. Therefore, the aims of this scoping review were to determine: (1) the specific protocols and modelling methods that have been implemented to quantify CS and *D*′ in running; (2) the deterministic potential of CS and *D*′ for running race performance; and (3) the application of CS and *D*′ in individualising running training prescription.

## Methods

### Design

This scoping review followed the guidelines set by the PRISMA Extension for Scoping Reviews [17]. The review protocol was registered on the Open Science Framework (OSF) prior to the commencement of database searches at: https://osf.io/rq2h3.

### Information Sources and Search Strategy

To identify all available studies investigating CS and *D*′ in a running context, a systematic search was conducted in PubMed, Scopus, Embase and SPORTDiscus on 1 December 2025. Reference lists of relevant articles were searched for additional relevant literature. No limits or filters were applied to the search, including date limits. The search strategy was conducted using a list of key search terms that were identified and agreed upon by the authors and organised into a Boolean search strategy: “runner*” OR “running” AND “distance prime” OR “finite work capacity” OR “finite distance capacity” OR “anaerobic running capacity” OR “anaerobic capacity” OR “critical speed” OR “critical velocity” OR “critical power” (see supplementary file (Electronic Supplementary Material) for the Boolean search strategy for each database).

### Eligibility Criteria

Study inclusion criteria were as follows: (1) human subjects only, (2) running-based context only, (3) aims to compare the CS test to another physiological threshold which implies similar physiology (e.g., maximal lactate steady-state), or another physiological threshold that approximates the heavy-severe intensity domain boundary demarcation, (4) aims to validate CS as the heavy-severe intensity domain boundary, (5) aims to compare CS or *D*′ derived from different testing protocols (e.g., different trial lengths) or conditions (treadmill, track, GPS, different environmental conditions), (6) aims to assess the reliability of the test, (7) compares the different modelling methods on the CS or *D*′ obtained, and/or determines the validity of the model against a performance test, (8) aims to determine the validity of the methods or modelling used to quantify *D*′ depletion or replenishment, (9) aims to assess the association between CS or *D*′ and World Athletics distance running events’ performance, (10) must be a study which has directly applied CS and *D*′ concepts in training (i.e., prescribing relative to CS, *D*′ depletion or time to exhaustion) inclusive of acute lab-based studies intending to replicate a training session.

Study exclusion criteria were as follows: (1) non-running modalities, (2) does not define the parameter as CS (e.g., maximal lactate steady state or second ventilatory threshold), (3) does not specifically use *D*′ (i.e., the physiological variable must be described as the work performed above CS or similar, not as ‘anaerobic capacity’), (4) reviews, abstracts and non-peer-reviewed sources, (5) studies not written in English, (6) full text unavailable.

Terms such as anaerobic distance capacity and anaerobic running capacity were observed in pilot searching to describe *D*′, typically due to also containing terms synonymous with CS in the study. However, in order to separate studies measuring *D*′ compared to anaerobic capacity from alternate tests (e.g., maximal accumulated oxygen deficit or running-based anaerobic sprint test), the ‘anaerobic capacity’ measure needed to be described as the work performed above critical speed or similar, or was derived from CS modelling.

Additionally, running CP and *W*′ were included. Running CP has been shown to occur at a comparable internal and external intensity to CS, as measured by $\dot{V}$O_2_ consumption [18] and running power [19], respectively. However, instances where running CP have been investigated have been described as CP throughout the review to highlight where studies measured CP instead of CS. As such, studies investigating CP derived from sensors have been included. Furthermore, CS estimated from training data has been included.

### Study Screening

Database search results were exported into EndNote (v21.0, Clarivate Analytics, Philadelphia, PA, USA) and subsequently uploaded to Covidence (Veritas Health Innovation, Melbourne, VIC, Australia) for screening, where duplicates were removed. The eligibility criteria were used for screening articles for inclusion. Two independent reviewers conducted title and abstract screening, followed by full-text screening of eligible articles. Discrepancies were resolved by discussion between the two screeners, with an additional reviewer being consulted if agreement was not reached.

### Data Extraction

A data extraction form was created in Excel (Microsoft Corporation, Albuquerque, NM, USA) to extract relevant data with the charting fields being agreed upon by the research team. Charting fields were chosen based upon items of interest related to research questions and were refined and added to based upon known studies prior to the search. A second reviewer cross-checked data from 20% of the studies to ensure accuracy [20]. The feedback from the second reviewer was discussed between the two reviewers and minor amendments were made to the data if any discrepancies arose. Data extracted included author and publication details, participant characteristics, the methods used to obtain CS and *D*′ (i.e., testing protocols and models used to derive CS and *D*′), the focus of the article (which included measurement validation and reliability, energetic modelling, relationship to exercise performance, and application of CS and *D*′ concepts; studies may have several purposes), and the terms used to describe CS and *D*′.

### Synthesis of Results

A PRISMA 2020 flow diagram was generated to depict the number of studies included or excluded throughout the review process. Studies investigating the measurement of CS were categorised into three groups: (1) studies assessing CS as the maximal metabolic steady state (MMSS), (2) studies assessing CS and *D*′ measurement, and (3) studies assessing CS measurement only. Some studies were assigned to multiple groups. The agreement between CS and other physiological thresholds for the MMSS has been reported in accordance with the results for each study, whereby CS being greater than or less than another physiological threshold is denoted by statistically significant differences. Studies that investigated modelled or predicted performance compared to actual performance were added to the CS and *D*′ measurement group, given that the modelling requires a CS and *D*′ estimate. Studies investigating the correlation between CS, *D*′ and *D*′ balance (the amount of *D*′ available for use at a given point within a race) and performance in running events have been summarised.

## Results

**Fig. 1:**
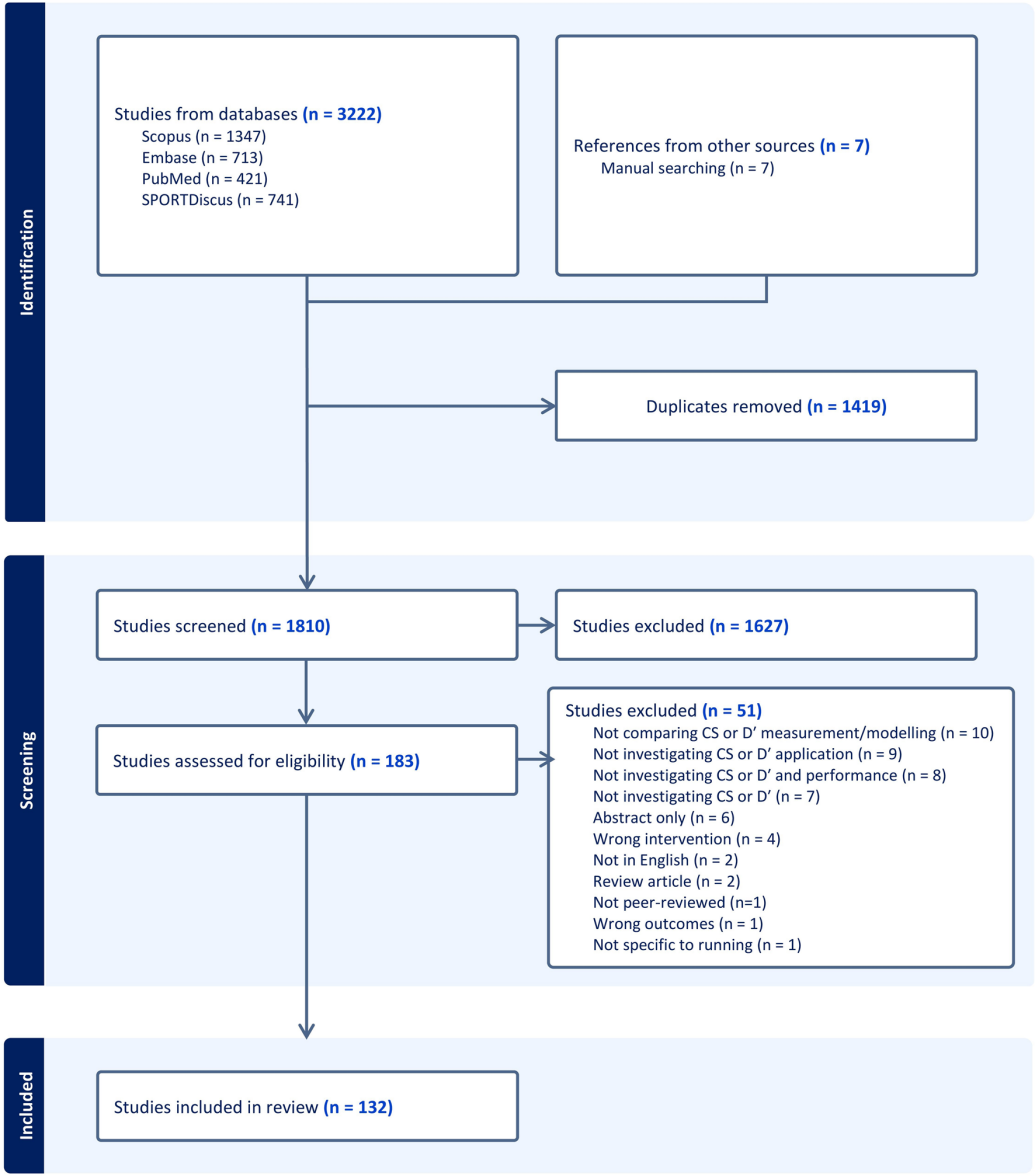
PRISMA (Preferred Reporting Items for Systematic reviews and Meta-Analyses) flow diagram for the selection of studies

The database search found 3222 records and a further seven were identified from manual searching. After removal of duplicates, 1810 articles were screened for title and abstract, and 183 full-text studies were subsequently assessed. 132 studies were included in the review (Fig. 1).

### CS and *D*′ Measurement and Modelling

Of the included studies, 25 investigated CS as the MMSS (Table 1). CS measurement was compared to the maximal lactate steady state (MLSS; *n* = 5), intermittent MLSS (*n* = 1), the second lactate threshold (LT2; *n* = 6), the second ventilatory threshold (VT2; *n* = 3), the onset of blood lactate accumulation (OBLA; *n* = 2), respiratory compensation point (RCP; *n* = 3), and lactate minimum velocity (LMV; *n* = 3). CS was assessed as the boundary of the heavy-severe domain via comparison of sub-, or supra-CS intensities (*n* = 7). Fundamentally, CS has been repeatedly validated as the heavy-severe exercise intensity domain transition; however, CS is not necessarily equivalent to physiological thresholds related to the MMSS. It is worth noting that the testing procedures and estimation techniques for CS, LT2, VT2, OBLA, RCP and LMV were not consistent between studies, which can impact parameter estimation (refer to Sect. 4.1).

**Table 1:** Studies assessing CS as the maximal metabolic steady-state, and comparison with previously established measures

	Study	Participant characteristics	Comparison of speed or power (CS vs. other physiological threshold)	Bias and limits of agreement	Finding
MLSS	de Lucas et al. [103]	8 endurance-trained male runners	15.2 ± 1.0 vs. 14.4 ± 0.6 km/h; *p* < 0.05	− 0.7 [− 2.2, 0.8] km/h	CS > MLSS
	Denadai et al. [104]	12 professional elite male soccer players	14.4 ± 1.1 vs. 13.1 ± 1.3 km/h; * p* < 0.05	–	CS > MLSS
	Motoyama et al. [105]	14 healthy males	10.2 ± 1.7 vs. 9.5 ± 1.2 km/h; * p* < 0.05	− 0.9 [− 2.4, 0.7] km/h	CS > MLSS
	Nixon et al. [106]	10 well-trained male runners and triathletes; $\dot{V}$O_2peak_ 63.0 ± 4.0 mL/kg/min	16.4 ± 1.3 vs. 15.2 ± 1.0 km/h; * p* < 0.001	–	CS > MLSS
	Smith and Jones [107]	8 recreationally active males; $\dot{V}$O_2max_ 54.9 ± 3.2 mL/kg/min	14.4 ± 1.1 vs. 13.8 ± 1.1 km/h; * p* > 0.05	0.6 [− 1.6, 2.8] km/h	CS = MLSS
Int. MLSS	de Lucas et al. [103]	8 endurance-trained male runners	15.2 ± 1.0 vs. 15.3 ± 0.7 km/h; * p* < 0.05	0.1 [− 0.9, 1.1] km/h	CS = int. MLSS
2nd lactate threshold	de Almeida et al. [108]	17 amateur runners; $\dot{V}$O_2max_ 58.38 ± 7.22 mL/kg/min	15.9 ± 2.1 vs. 15.9 ± 1.2 km/h; * p* > 0.05	–	CS = LT2
	Guimaraes and Da Silva [109]	19 male runners	13.7 ± 1.3 vs. 13.8 ± 1.4 km/h; * p* = 0.215	0.1 [− 0.3, 0.5] km/h	CS = LT2
	Kalva-Filho et al. [83]	14 futsal players; $\dot{V}$O_2peak_ 41.0 ± 8.9 mL/kg/min	10.1 ± 1.1 vs. 9.4 ± 0.7 km/h; * p* = 0.01	− 0.7 [− 2.5, 1.0] km/h	CS > LT2
	Silva et al. [110]	11 physical education students; $\dot{V}$O_2max_ 48.9 ± 5.8 mL/kg/min	12.0 ± 1.8 vs. 11.1 ± 1.7 km/h; * p* < 0.001	–	CS > LT2
	Smith and Jones [107]	8 recreationally active males; $\dot{V}$O_2max_ 54.9 ± 3.2 mL/kg/min	14.4 ± 1.1 vs. 13.7 ± 0.6 km/h; * p* > 0.05	0.7 [− 2.0, 3.4] km/h	CS = LT2
	Striegel et al. [111]	30 males; 10 untrained, 10 runners, 10 400 m runners	*p* < 0.001	–	CS > LT2
VT2	Dearing and Paton [91]	16 males, 4 females; 2 internationally competitive, 3 national class, 15 recreational runners; 39.5 ± 14.6 years old, $\dot{V}$O_2peak_ 3.93 ± 0.73 L/min	302 ± 58 vs. 304 ± 65 W; * p* > 0.05	–	CS = VT2
	Ruiz-Alias et al. [112]	14 athletes; $\dot{V}$O_2max_ 66.3 ± 7.2 mL/kg/min	4.72 ± 0.42 vs. 3.88 ± 0.42 W/kg; * p* > 0.05	–	CS > VT2
	Ruiz-Alias et al. [113]	10 males, 9 females; recreational runners; $\dot{V}$O_2max_ 58.4 ± 5.3 and 53.0 ± 4.7 ml/kg/min, respectively	Males: 312 ± 44 vs. 305 ± 41 W; * p* = 0.130 Females: 214 ± 20 vs. 213 ± 21 W; * p* = 0.168	–	CS = VT2
OBLA	Dearing and Paton [91]	16 males, 4 females. Two internationally competitive, 3 national class, 15 recreational runners; 39.5 ± 14.6 years old, $\dot{V}$O_2peak_ 3.93 ± 0.73 L/min	302 ± 58 vs. 296 ± 56 W; * p* > 0.05	–	CS = OBLA
	Denadai et al. [104]	12 professional elite male soccer players	14.4 ± 1.1 vs. 13.6 ± 1.4 km/h; * p* > 0.05	–	CS = OBLA
RCP	Broxterman et al. [114]	40 males, 30 females	11.7 ± 2.3 vs. 11.5 ± 2.3 km/h; * p* = 0.208	–	CS = RCP
	Massini et al. [115]	12 male runners; $\dot{V}$O_2max_ 53.0 ± 5.1 ml/kg/min	Speed: NR; * p* = 0.92 %VO_2max_: 89.6 ± 3.1 vs. 90.2 ± 2.8%; * p* = 0.84	0.1 [− 0.8, 1.0] km/h 0.4 [− 2.0, 2.7] ml/kg/min	CS = RCP
	Ruiz-Alias et al. [112]	14 athletes; $\dot{V}$O_2max_ 66.3 ± 7.2 mL/kg/min	4.7 ± 0.4 vs. 4.6 ± 0.4 W/kg; * p* = 0.184	–	CS = RCP
LMV	Browne et al. [116]	18 males, 7 females; middle- and long-distance youth runners	13.5 ± 1.9 vs. 13.3 ± 1.3 km/h; * p* = 0.305	0.2 [− 1.7, 2.1] km/h	CS = LMV
	Camargo et al. [117]	12 females; well-trained basketball players	10.3 ± 0.2 vs. 9.5 ± 0.1 km/h; * p* < 0.05	–	CS > LMV
	Simões et al. [92]	20 male endurance runners	17.5 ± 1.1 vs. 16.9 ± 0.9 km/h; * p* < 0.001	–	CS > LMV
Heavy-severe boundary validation	Broxterman et al. [73]	4 men, 3 women; $\dot{V}$O_2max_ 49.6 ± 5.7 mL/kg/min	–	–	CS is the H–S boundary
	Bull et al. [90]	6 males, 4 females; $\dot{V}$O_2max_ 51 ± 6 mL/kg/min	–	–	Mixed results. All models developed a $\dot{V}$O_2_ slow component except for the exponential model. No models reached $\dot{V}$O_2max_. 3-parameter hyperbolic model had a lower peak $\dot{V}$O_2_ and HR than other models
	Follador et al. [96]	31 men, 11 women. Recreational runners. $\dot{V}$O_2max_ 52.5 ± 6.6 mL/kg/min	–	–	CS is the H–S boundary
	Hill and Ferguson [118]	5 men, 7 women. $\dot{V}$O_2max_ 52.7 ± 14.5 mL/kg/min	–	–	CS is the H–S boundary
	Hill et al. [30]	18 men, 7 women. Kinesiology students	–	–	CS is the H–S boundary
	Kirby et al. [98]	10 males, 2 females. Recreationally active adults	–	–	CS is the H–S boundary
	Nixon et al. [106]	10 well-trained male runners and triathletes; $\dot{V}$O_2peak_ 63.0 ± 4.0 mL/kg/min	–	–	CS is the H–S boundary

*CS* critical speed, *H–S* heavy-severe, *int. MLSS* intermittent maximal lactate steady-state, *LMV* lactate minimum velocity, *LT2* the second lactate threshold, *MLSS* maximal lactate steady-state, *OBLA* onset of blood lactate accumulation, *RCP* respiratory compensation point, $\dot{V}$*O*_*2max*_ maximal oxygen consumption, $\dot{V}$*O*_*2peak*_ peak oxygen consumption

Seventy studies investigated the testing protocols and modelling methods for estimating CS and *D*′ (Table 2), and 18 investigated the testing protocols and modelling methods for CS measurement only (Table 3). Tables 2 and 3 detail variations in test distance, duration and/or intensity, the subsequent modelling procedure, and the reason for study inclusion (i.e., the assessment and comparison of testing protocols and modelling). A wide range of testing methods (and subsequent modelling) was used to assess CS and *D*′ estimation. Twenty-two studies compared CS and *D*′ estimates derived from different models [13, 15, 21–40] (Table 4). Twenty-three studies compared actual and predicted performance from CS and *D*′ estimates, or from different models [13, 23–25, 28, 34, 37, 38, 41–55]. Twenty-five studies investigated the influence of different testing protocols or conditions (e.g., the number of trials used, the length of trials, surface type, the effect of ambient conditions) [21, 28, 38, 41, 53–72]. Eight studies compared multi-trial testing to the 3-min test (3MT) [14, 26, 27, 42, 60, 73–75]. Six studies investigated the measurement of intermittent CS [56, 76–80]. Twelve studies assessed the reliability of CS and *D*′ testing protocols [26, 42, 44, 60, 75, 78, 81–86] (Table 3). Furthermore, six studies investigated *D*′ reconstitution (Table 5) [3, 7, 80, 87–89]. For CS (-only) measurement studies, four studies compared CS estimates derived from different models [43, 90–92]. Fourteen studies investigated the influence of different testing protocols or conditions [18, 92–102]. Four studies assessed the reliability of CS testing protocols [96, 98, 100].

**Table 2:** Testing protocol characteristics and modelling methods of studies investigating critical speed (CS) and *D*′ measurement

Study	Alternative test protocol	Fixed effort distance	Fixed effort duration	Fixed effort intensity	linear (distance time)	linear (inverse time)	2-parameter hyperbolic	3-parameter hyperbolic	Exponential	Other models	Measurement or modelling assessment
Ari and Deliceoglu [15]	–	2 trials; 800 and 2400 m	–	–	x	x	x	–	–	–	Comparison of models for determining CS and * D*′ values
Ari et al. [21]	–	–	3 trials; 6, 9, 12 min	–	x	x	–	–	–	–	Comparison of models for determining CS and * D*′ values; comparing two different COD test protocols on CS and * D*′
Beck et al. [22]	–	–	–	4 trials; 1–12 min	x	x	x	–	–	–	Comparison of models for determining * D*′ values
Berthoin et al. [76]	–	–	–	3 continuous trials at 90, 100, 110% MAV. 3 intermittent trials (15:15 s) at 120, 130, 140% MAV with passive recovery	x	–	–	–	–	–	A comparison between continuous and intermittent CS and * D*′
Billat et al. [23]	–	–	–	4 trials; 90, 100, 120, 140% v$\dot{V}$O_2max_	–	–	x	x	–	–	Comparison of models for determining CS and * D*′ values; comparison of models for predicting performance
Bosquet et al. [24]	–	–	–	5 trials; 95, 100, 105, 110, 120% PTV	x	x	x	x	x	–	Comparison of models for determining CS and * D*′ values; comparison of models for predicting performance
Broxterman et al. [73]	3MT	–	–	3 trials; 90, 100, 120% Speak	–	x	–	–	–	–	Comparison between multi-trial testing and 3MT for determining CS and * D*′ values
Buchheit et al. [56]	–	–	–	3 intermittent trials (at 15:15 and 30:30 s each) at 90, 95, 100% VIFT	x	–	–	–	–	–	Comparison of testing protocols for determining intermittent CS and * D*′ values
Busso and Chatagnon [41]	–	8 distances^a^; 800, 1000, 1500, 1600, 2000, 3000, 5000, 10,000 m	–	–	–	–	x	–	–	–	Comparison of distances used within the model for determining CS and * D*′ values. Comparison between predicted and actual performance
Busso et al. [42]	3MT	2 trials; 1200, 3600 m	–	–	x	–	–	–	–	–	Comparison between multi-trial testing and 3MT for determining CS and * D*′ values. Reliability of 3MT. Performance prediction based on CS and * D*′ estimates
Çabuk et al. [25]	–	3 trials^a^; 1500, 3000, 5000 m	–	–	x	x	x	–	–	–	Comparison of models for determining CS and * D*′ values; comparison of models for predicting performance
Carter and Dekerle [57]	–	–	–	4 trials; 80% difference of LT and $\dot{V}$O_2max_, 100, 110 and 120% $\dot{V}$O_2max_	–	x	–	–	–	–	A comparison of CS and * D*′ determined under different test conditions (flat vs. gradient)
de Aguiar et al. [26]	3MT	3 trials; 800, 1600, 2400 m	–	–	x	x	–	–	–	–	Comparison between models, and 3MT for determining CS and * D*′ values. Reliability of 3MT
Fukuda et al. [78]	–	–	–	3 intermittent trials (10:10 s) at 110, 120, 130% PV	x	–	–	–	–	–	Reliability assessment. Parameter assessment (critical rest interval)
Fukuda et al. [77]	–	–	–	3 intermittent trials (15:15 s) at 110, 120, 130% PV	x	–	–	–	–	–	Parameter assessment (critical rest interval)
Galbraith et al. [81]	–	3 trials; 1200, 2400, 3600 m	–	–	x	–	–	–	–	–	Reliability assessment
Galbraith et al. [58]	–	3 trials; 1200, 2400, 3600 m	–	3 trials; 100, 105, 110% v$\dot{V}$O_2max_	x	–	–	–	–	–	A comparison of protocols for determining CS and * D*′ values (same-day track TTs [different recovery periods] vs. multi-visit treadmill TTEs)
Gama et al. [27]	3MT	–	–	4 trials; varying resistances by elastic cords	x	x	x	–	–	–	Comparison between models, and 3MT for determining CS and * D*′ values
Gama et al. [82]	3MT	–	–	–	–	–	–	–	–	–	Reliability assessment
Gamelin et al. [43]	–	–	3 trials; 6, 9, 12 min	–	x	x	x	x	x	–	Comparison of models for predicting performance
Gifford and Collins [59]	–	3 trials^a^; 800, 1500, 3000 m	–	–	x	–	–	–	–	–	Comparison of number of trials (2 vs. 3) for determining CS and * D*′ values
Girardi et al. [28]	–	3000, 5000, 10,000 m, half- and full-marathon^a^	–	–	x	–	x	–	–	–	Comparison of distances used within the model for determining CS and * D*′ values; comparison of models for determining CS and * D*′ values; comparison of models for predicting performance
Hill et al. [29]	–	–	–	5–7 trials; ~ 3 to ~ 10 min	x	x	x	x	x	–	Comparison of models for determining CS and * D*′ values
Hill et al. [30]	–	–	–	5 trials; ~ 2.5 to 16 min	x	x	x	–	–	–	Comparison of models for determining CS and * D*′ values
Hinckson and Hopkins [44]	–	–	–	3 trials; ~ 2, 4 and 8 min	x	–	–	–	–	–	Reliability of testing results. Performance prediction based on modelling
Housh et al. [31]	–	–	–	4 trials; ~ 2–12 min	x	x	x	x	x	–	Comparison of models for determining CS and * D*′ values
Hughson et al. [45]	–	–	–	6 trials; 19.2–22.4 km/h (~ 2–12 min)	–	x	–	–	–	–	Performance prediction
Hunter et al. [60]	3MT	–	3 trials; 3, 7, 12 min	–	x	x	x	–	–	–	Comparison of CS and * D*′ derived from training, TTs and a 3MT. Measurement reliability
Jaén-Carrillo et al. [72]	–	–	2 trials; 3 and 9 min	–	–	x	–	–	–	–	Comparison of surface (track, road and, gravel) on CS and * D*′ estimation
Kachouri et al. [79]	–	–	–	2 continuous trials at 95 and 105% vMTT. 2 intermittent trials at 95 and 105% vMTT (1:1 @ half of Tlim (continuous) of the respective intensity. Jog recovery)	x	–	–	–	–	–	Comparison of continuous and intermittent CS and * D*′
Kalva-Filho et al. [83]	3MT (20 m shuttle)	–	–	–	–	–	–	–	–	–	Reliability assessment
Kordi et al. [61]	–	3 trials; 1200, 2400, 3600 m	–	–	x	–	–	–	–	–	Comparison of the number of trials used (2 vs. 3) for determining CS and * D*′ values
Kramer et al. [62]	3MT (traditional, and 50 and 25 m shuttle)	–	–	–	–	–	–	–	–	–	Comparison of linear and shuttle 3MT CS and * D*′ values
Kramer et al. [63]	3MT (traditional, and 50 and 25 m shuttle)	–	–	–	–	–	–	–	–	Bi-exponential modelling	Comparison of linear and shuttle 3MT CS and * D*′ values. Comparison of values using typical method and modelling
Kramer et al. [64]	3MT (traditional, and 50 and 25 m shuttle)	–	–	–	–	–	–	–	–	–	Comparison of linear and shuttle 3MT CS and * D*′ values
Kuo et al. [84]	3MT	–	–	–	–	–	–	–	–	–	Reliability assessment
Lanzarini et al. [40]	1	3 trials; 800, 1500, 3000 m	–	–	x	x	x	x	–	–	Comparison of models for determining CS and * D*′ values
Leslie et al. [65]	–	–	–	4 trials; ~ 2–15 min	–	x	–	–	–	–	Comparison of CS and * D*′ values under different environmental conditions
Liu et al. [119]	3MT (30 m shuttle)	–	–	–	–	–	–	–	–	Bi-exponential modelling	A comparison of CS and * D*′ values from typical 3MT interpretation vs. bi-exponential modelling
Loures et al. [32]	–	–	–	100, 110 and 120% v$\dot{V}$O_2max_	x	x	–	–	–	–	Comparison of models on * D*′ value
Manuel and Lance [46]	–	100 m, 200 m, 400 m, 800 m, 1000 m, 1500 m, 1609 m (1 mile), 2000 m, 3000 m, 5 km (all surfaces), 10 km (all surfaces), 1-h run, 21.1 km (half-marathon), 42.2 km (marathon)^a^	–	–	x	–	–	–	–	–	Performance prediction
Massini et al. [66]	–	3 trials; 900, 2100, 3300 m	–	3 trials; 90, 95, 115% v$\dot{V}$O_2max_	x	x	–	–	–	–	Comparison of trial surface, and TT vs. TTE tasks on CS and * D*′ values
Mizelman et al. [33]	–	–	–	–	–	x	–	x	x	Omni-domain model	Comparison of CS and * D*′ between different models
Morton and Billat [80]	–	3000, 5000, 10,000 m^a^	–	3 intermittent trials; 60:60 s @ 120:50% CS; 180:180 s @ 110:60% CS; 30:60 s @ 135:65% CS	x	–	–	–	–	–	Comparison of continuous and intermittent CS and * D*′ values
Nimmerichter et al. [34]	–	–	–	3 trials; 70% of the difference between VT and MAS, 98, 110% MAS	x	x	x	–	–	–	A comparison of models for CS and * D*′ estimation. Comparison of models for performance prediction
Nimmerichter et al. [85]	–	3 trials; 500, 1200, 2000 m	–	–	–	x	–	–	–	–	Reliability assessment
Patoz et al. [35]	–	–	–	4 trials; 90, 100, 110, 120% peak speed	–	x	x	x	x	–	Comparison of models on CS and * D*′ values
Patoz et al. (2021) [36]	–	–	–	4 trials; 90, 100, 110, 120% peak speed	x	x	x	–	–	–	Comparison of models on CS and * D*′ values
Pepper et al. [47]	–	–	–	4 trials; 12.88–21.74 km/h	x	–	x	–	–	–	Performance prediction
Perez et al. [48]	3MT	–	–	–	–	–	–	–	–	–	Performance prediction
Pettitt et al. [49]	3MT	–	–	–	–	–	–	–	–	–	Performance prediction
Ruiz-Alias et al. [50]	–	–	4 trials; 3, 5, 10, 20 min	–	–	x	–	–	–	–	Comparison of predicted and actual performance. Variability of estimates
Ruiz-Alias et al. [86]	–	–	2 trials; 3, 9 min	–	–	x	–	–	–	–	Reliability assessment
Ruiz-Alias et al. [67]	–	–	2 trials; 3, 9 min	–	–	x	–	–	–	–	Effect of different surfaces, trial order (long-short or short-long), and same-day vs. multi-visit testing on CP and * W*′
Ruiz-Alias et al. [13]	–	–	4 trials; 3, 5, 10, 20 min	–	x	x	–	x	–	Stryd and Golden cheetah	Comparison of models for determining CS and * D*′ values; comparison of models for predicting performance
Ruiz-Alias et al. [37]	–	–	6 trials; 3, 4, 5, 10, 20, 30 min	–	x	x	x	x	–	Stryd and Golden cheetah	Comparison of models for determining CS and * D*′ values; comparison of models for predicting performance
Ruiz-Alias et al. [38]	–	–	6 trials; 3, 4, 5, 10, 20, 30 min	–	x	x	x	x	–	Stryd and Golden cheetah	Comparison of models, task lengths used, and number of trials used on CP and * W*′. Performance prediction
Ruiz-Alias et al. [51]	–	–	5 trials; 3, 4, 5, 10, 20 min	–	–	x	–	–	–	–	Performance prediction based on CP vs. CS modelling
Saari et al. [14]	3MT (30 m shuttle)	3 trials; 600, 810, 1020 m	–	–	x	x	–	–	–	–	Comparison of multi-trial testing to 3MT shuttle on CS and * D*′ values
Smith et al. [68]	–	–	–	4 trials; 90, 100, 105, 110% PV	x	–	–	–	–	–	Comparison of number of trials required, and the lengths of the trials used on CS and * D*′ values
Smyth et al. [52]	–	–	–	–	x	–	–	–	–	–	Performance prediction (from training data)
Smyth and Muniz-Pumares [53]	–	–	–	–	x	–	–	–	–	–	Comparison of different distances modelled on CS and * D*′ values. Performance prediction
Solomonson et al. [69]	3MT	–	–	–	–	–	–	–	–	–	Effect of external loading on CS and * D*′ values
Sousa et al. [74]	3MT	–	–	3 trials; 2–10 min	x	x	–	–	–	–	Comparison of 3MT and multi-trial testing on CS and * D*′ values
Triska et al. [70]	–	–	2, 5, 10 and 3, 7, 12 min	–	–	x	–	–	–	–	Comparison of task lengths tested on CS and * D*′ values
Triska et al. [71]	–	–	Duration matched to treadmill trials	75% difference of VT and MAS, 98%, 108% MAS	–	x	–	–	–	–	Influence of surface type, and TT vs. TTE on CS and * D*′ values
Vandewalle [54]	–	1500, 3000, 5000, 10,000 m^a^	–	–	–	–	x	x	x	–	Comparison of distances modelled on CS and * D*′ values. Performance prediction of models
Vassallo et al. [75]	3MT	–	–	70% of the difference between GET and v$\dot{V}$O_2max_, 80% of the difference between GET and v$\dot{V}$O_2max_, 100, 110% v$\dot{V}$O_2max_	x	–	–	–	–	–	Multi-visit testing vs. 3MT for CS and * D*′ values. Reliability assessment
Zagatto et al. [39]	–	–	–	3 trials; 100, 110, 120% v$\dot{V}$O_2max_	x	x	x	–	–	–	Comparison of models on CS and * D*′ values
Zinoubi et al. [55]	–	1500, 3000, 5000, 10,000 m^a^	–	3 trials; 90, 100 and 110% sMTT	x	–	–	–	–	–	Comparison of trial lengths on CS and * D*′ values. Performance prediction

*CP* critical power, *CS* critical speed, *CVST* critical velocity shuttle test, *GET* gas exchange threshold, *LT* lactate threshold, *MAS* maximal aerobic speed, *MAV* maximal aerobic velocity, *PTV* peak treadmill velocity, *PV* peak velocity, *sMTT* speed of Montreal track test, *Speak* peak speed, *TT* time trial, *TTE* time to exhaustion, *VIFT* variable intermittent fitness test, *vMTT* velocity of Montreal track test, *VT* ventilatory threshold, *v*$\dot{V}$*O*_*2max*_ velocity of $\dot{V}$O_2max_, – no data to report, *x* the model used

^a^Race data

**Table 3:** Testing protocol characteristics and modelling methods of studies investigating critical speed (CS) and *D*′ measurement

Study	Alternate test protocol	Fixed effort distance	Fixed effort duration	Fixed effort intensity	linear (distance time)	linear (inverse time)	2-parameter hyperbolic	3-parameter hyperbolic	Exponential	Stryd	Measurement or modelling assessment
Antonio et al. [93]	T10	2 trials; 1200, 2400 m	–	–	x	–	–	–	–	–	Comparison of multi-trial testing vs. T10 on CS value
Barbosa et al. [120]	T10	3 trials; 1200, 2400, 3600 m	–	–	x	–	–	–	–	–	Comparison of multi-trial testing vs. T10 on CS value. Reliability assessment
Bull et al. [90]	–	–	–	4–5 trials; lasting ~ 3–20 min	x	x	x	x	x	–	Comparison of models on CS value
de Borba et al. [94]	T10	3 trials; 1200, 2400, 3600 m	–	–	x	–	–	–	–	–	Comparison of multi-trial testing vs. T10 on CS value
Dearing and Paton [91]	–	–	4 trials; 3, 5, 10, 20 min	–	–	x	–	–	–	x	Comparison of models on CS value
Follador et al. [96]	T10	3 trials; 1200, 2400, 3600 m	–	–	x	–	–	–	–	–	Comparison of multi-trial testing vs. T10 on CS value. Reliability assessment
Figueiredo et al. [95]	–	3 trials; 1000, 1800, 2600 m	–	–	x	–	–	–	–	–	Comparison of number and length of trials used on CS value
Gorostiaga et al. [97]	–	1000, 1500, 3000, 5000, 10,000 m, half marathon, and marathon^a^	–	–	x	–	–	–	–	–	Comparison of number and length of trials used on CS value
Jaén-Carrillo et al. [121]	–	–	2 trials; 3 and 9 min	–	–	x	–	–	–	–	Comparison of task surface (track vs. trail) on CS estimation
Kirby et al. [98]	CVST	3 trials; 1200, 2400, 5000 m	–	–	x	–	–	–	–	–	Comparison of multi-trial testing vs. CVST on CS value. Reliability assessment
Kranenburg and Smith [99]	–	3 trials; 907, 2268, 4082 m	–	3 trials; ~ 3, 7, 13 min	x	x	–	–	–	–	Comparison of testing methods on CS (track TT vs. treadmill TTE)
Lord et al. [100]	–	Shuttle running test; 100, 400, 1500 m 3 trials; 1200, 2400, 3600 m	1, 5, 10, 60, 300 s	–	x	x	–	–	–	–	Comparison multi-trial testing vs. gameplay data on CS value. Reliability assessment
Ñancupil-Andrade et al. [101]	–	–	10 trials; 1, 2, 3, 4, 5, 10, 20, 30, 50, 60 min	–	–	–	–	–	–	x	Comparison of methods on CS value (TTs vs. 95% 20 min TT)
Olaya–Cuartero et al. [102]	–	Half marathon^a^	3, 9 min	–	–	–	–	–	–	x	Comparison of methods on CS value (TTs vs. half marathon power)
Patoz et al. [19]	–	–	–	90, 100, 110, 120% peak speed	–	–	–	x	–	–	Comparison of different CP values and CS
Gamelin et al. [43]	–	–	6, 9, 12 min	–	x	x	x	x	x	–	Comparison of models on CS value
Simões et al. [92]	–	2 trials; 500, 3000 m	–	Heart rate deflection point velocity	x	x	–	–	–	–	Comparison of 2 vs. 3 trials and model on CS value
van Rassel et al. [18]	–	3 trials; 1200, 2400, and 3600, 4000 or 4400 m	–	–	–	–	x	–	–	–	Comparison of CP and CS and 2 vs. 3 trials on CS value

*COD* change of direction, *CP* critical power, *CS* critical speed, *TT* time trial, *TTE* time to exhaustion, – no data to report, *x* the model used

^a^Race data used

**Table 4:** Summary of typical model critical speed (CS) and *D*′ bias, standard error ranges and the minimum data requirements to derive CS and *D*′ estimates

Model	CS and * D*′ bias	Typical standard error range	Minimum data requirements
Distance-time	CS: likely comparable to inverse-time and 2-parameter hyperbolic models [13, 15, 21, 24, 27–31, 35–37, 116]. Likely greater than 3-parameter hyperbolic model [13, 23, 24, 29, 31, 33, 35, 37]. Variable difference to exponential model [24, 29, 31, 35] *D*′: likely comparable to inverse-time and 2-parameter hyperbolic models [13, 15, 21, 24, 27–31, 35–37, 116]. Likely lower than 3-parameter hyperbolic model [13, 23, 24, 29, 31, 33, 35, 37].	CS: 1–2.8% [29–31, 34, 90] *D*′: 5–28.2% [29, 30, 34, 39]	2 trials of different durations
Inverse-time	CS: likely comparable to distance-time and 2-parameter hyperbolic models [13, 15, 21, 24, 27–31, 35–37, 116]. Likely greater than 3-parameter hyperbolic model [13, 23, 24, 29, 31, 33, 35, 37]. Variable difference to exponential model [24, 29, 31, 35] *D*′: likely comparable to distance-time and 2-parameter hyperbolic models [13, 15, 21, 24, 27–31, 35–37, 116]. Likely lower than 3-parameter hyperbolic model [13, 23, 24, 29, 31, 33, 35, 37].	CS: 2.3–3.8% [29–31, 34, 90] *D*′: 7–20.5% [29, 30, 34, 39]	2 trials of different durations
2-parameter hyperbolic	CS: likely comparable to distance-time and inverse-time models [13, 15, 21, 24, 27–31, 35–37, 116]. Likely greater than 3-parameter hyperbolic model [13, 23, 24, 29, 31, 33, 35, 37]. Variable difference to exponential model [24, 29, 31, 35] *D*′: likely comparable to distance-time and inverse-time models [13, 15, 21, 24, 27–31, 35–37, 116]. Likely lower than 3-parameter hyperbolic model [13, 23, 24, 29, 31, 33, 35, 37].	CS: 1.8–3% [29–31, 34, 90] *D*′: 6–23.3% [29, 30, 34, 39]	2 trials of different durations
3-parameter hyperbolic	CS: Likely less than distance-time, inverse-time, and 2-parameter hyperbolic model [13, 23, 24, 29, 31, 33, 35, 37]. Variability in difference to exponential model *D*′: Likely greater than distance-time, inverse-time, and 2-parameter hyperbolic model [13, 23, 24, 29, 31, 33, 35, 37]	CS: 16.9–70.9% [29, 31, 90] *D*′: > 100% [29]	2 trials of different durations
Exponential	Variable difference to distance-time, inverse-time, and 2- and 3-parameter hyperbolic models [24, 29, 31, 35]	CS: 7.7–9.6% [29, 31, 90]	2 trials of different durations
Stryd	CP: comparable to work-time model. Less than inverse-time model. Greater than 3-parameter hyperbolic model [13]	N/A	CP can be generated after a few runs [91]

*CP* critical power, *CS* critical speed

**Table 5:** Studies investigating *D*′ reconstitution and *D*′ balance modelling

Study	Equation for * D*′ balance or reconstitution equation	Work and recovery characteristics to derive * D*′ balance reconstitution or time constant	Derived time constant	Findings
Bellenger et al. [89]	*D*′ reconstitution to estimate * D*′ balance = *D*′ − (*D*′− *D*′_(ustart)_) × e^−*u*/*τD*′^	Intermittent running with passive recovery from training data (various sessions) used to derive time constant	362 ± 148 s	*D*′ balance modelling was able to differentiate exhaustive vs. non-exhaustive sessions Modelling characterised exhaustive sessions as exhaustive (in 75% of cases) when an individual time constant was used during sessions involving passive recovery A recovery intensity specific time constant is required for active recovery
Galbraith et al. [7]	Linear recovery: (CS − recovery speed) × recovery duration Non-linear recovery: Skiba et al. [2] integral equation	Three separate trials to exhaustion (1) 1000: 200 m at 107:95% CS (2) 600: 200 m at 110:90% CS (3) 200: 200 m at 150:80% CS	95%: 353 ± 118 s 90%: 378 ± 100 s 80%: 397 ± 167 s	Linear nor non-linear recovery models were more accurate for predicting time to exhaustion in intermittent running. Non-linear models tended to result in a negative * D*′ balance during exhaustive exercise Exhaustion predictions were less accurate for shorter, high-intensity intervals
Kirby et al. [87]	Skiba et al. [2] integral equation	60:30 s work:recovery. Work bout at an intensity to elicit exhaustion in 4 min. Three separate recovery conditions: (1) Moderate-intensity recovery: walking at 3.3 mph (2) Heavy-intensity recovery: 10% below the predicted CS (3) Severe-intensity recovery: calculated to be the velocity to elicit exhaustion in 12 min	NR	Average SmO_2_ rate over the duration of the work bout can predict TTE *D*′ balance can be estimated by the cumulative time spent at a given %SmO_2_
Morton and Billat [80]	(CS − recovery speed) × recovery duration	Used to model CS and * D*′ from intermittent running. Three separate trials to exhaustion (1) 60:60 s at 120:50% CS (2) 180:180 s at 110:60% CS (3) 30:60 s at 135:65% CS	N/A	CS and * D*′ estimations are modelled differently during intermittent exercise, suggesting recovery during work bouts
Vaccari et al. [88]	N/A	Three separate trials altering the duration of an initial work bout before a recovery period (1) 30 s work: 120 s recovery: until exhaustion. Performed at estimated TTE of 6 min: 66% CS: estimated TTE of 6 min (2) 120 s work: 120 s recovery: until exhaustion. Performed at estimated TTE of 6 min: 66% CS: estimated TTE of 6 min 3) until exhaustion: 120 s recovery: until exhaustion. Performed at estimated TTE of 6 min: 66% CS: estimated TTE of 6 min	N/A	The rate of * D*′ recovery is faster after a greater depletion of * D*′, suggesting an exponential reconstitution
Vassallo et al. [3]	Skiba et al. integral equation using cycling and running derived time constants [2] *W*′ balance differential equation	60:30 s work:rest. Four separate trials to exhaustion with different recovery intensity conditions (1) Light-domain recovery at 40% v$\dot{V}$O_2max_ (2) Moderate-domain recovery at 90% vGET (3) Heavy-domain recovery at vGET + 50% Δ2 (where Δ2 = CS − vGET) (4) Severe-domain recovery at v$\dot{V}$O_2max_ − 50% Δ1 (where Δ1 = v$\dot{V}$O_2max_ − CS)	Light: 119 ± 32 s Moderate: 190 ± 45 s Heavy: 336 ± 77 s Time constant fit for difference between recovery power and CP: 372e^(−0.02DCP)^ + 102	Different recovery intensities derive different recovery time constants, which occurs in a curvilinear relationship with the difference between the recovery intensity and CP Skiba et al.'s [2] integral equation, and differential equation accurately estimated *D*′ balance of 0 m at exhaustion when a running-specific time constant is solved for

*CP* critical power, *CS* critical speed, *DCP* difference between recovery intensity and CP, *D′*_*(ustart)*_ *D*′ balance at the start of the recovery segment, *e* Euler’s number, *N/A* not applicable, *NR* not reported, *SmO*_*2*_ muscle oxygen saturation, *τD′* time constant of *D*′ recovery, *TTE* time to exhaustion, *vGET* velocity at gas exchange threshold, *v*$\dot{V}$*O*_*2max*_ velocity at $\dot{V}$O_2max_

### Critical Speed (CS) and *D*′ as Determinants of Performance

Twenty-seven studies investigated the relationship between CS or *D*′ (or *D*′ balance) and running performance (Table 6). Distances investigated were 100 m (*n* = 1), 400 m (*n* = 2), 800 m (*n* = 7), 1000 m (*n* = 1), 1500 m (*n* = 4), 1600 m (mile; *n* = 1), 3000 m (*n* = 5), 5000 m (*n* = 9), 10,000 m (*n* = 9), and half (*n* = 3) and full marathon (*n* = 2).

**Table 6:** Studies that have investigated the correlation between critical speed (CS), *D*′ and *D*′ balance on performance

Distance	Study	Participant characteristics	Performance parameter vs. modelled parameter investigated	Correlation (*r* value; *p* value)	Summary per distance
100 m	Beck et al. [22]	13 Brazilian army men; 24.6 ± 6.6 years old	Time; * D*′	0.17; > 0.05	
400 m	Gifford and Collins [59]	Masters athletes (*n* = 24 for correlational analysis)	Time; CS, * D*′	CS: − 0.86; < 0.001 *D*′: 0.53; 0.035	A greater CS is associated with reduced time and increased speed Mixed findings for * D*′ showing a weak, and moderate association with speed and time, respectively
	Walt et al. [131]	French, Italian, Polish, Swiss and British national runners (851,647 season-best performances)	Speed; CS, * D*′	CS: 0.70; < 0.001 *D*′: 0.04; < 0.001	
800 m	Ari and Deliceoglu [15]	13 amateur soccer players; 22.7 ± 5.3 years old	Time: CS, * D*′	CS: − 0.45; 0.121 *D*′: − 0.77; 0.002	Mixed findings with CS not correlated to performance time in 3 studies, but a higher CS was associated with improved speed in 2 studies Mixed findings with performance time being faster with a larger * D*′ in 1 of 2 studies. * D*′ not correlated (or weakly associated) to speed, although race speed above CS increases as * D*′ increases
	Bellinger et al. [132]	20 male middle distance runners; 20.8 ± 2.5 years old, $\dot{V}$O_2max_ 69.7 ± 5.5 mL/kg/min	Performance time in fast and paced races: CS and * D*′	NR; > 0.05	
	Bosquet et al. [12]	17 middle- and long-distance runners; 23 ± 3 years old, $\dot{V}$O_2max_ 66.5 ± 7.3 mL/kg/min	Speed: * D*′	− 0.07; > 0.05	
	Bosquet et al. [24]	17 middle- and long-distance runners; 23 ± 3 years old, $\dot{V}$O_2max_ 66.5 ± 7.3 mL/kg/min	Speed: CS	0.68; < 0.05	
	Pettitt et al. [49]	14 collegiate female distance runners; 19 ± 1 years old, $\dot{V}$O_2max_ 55 ± 4 mL/kg/min	Race speed above CS: * D*′	0.99; NR	
	Pompeu Trevelin et al. [133]	20 male university athletes; 22.9 ± 3.0 years old, $\dot{V}$O_2max_ 46.2 ± 4.9 mL/kg/min	Time: CS	Only significant correlations reported *p* > 0.05	
	Walt et al. [131]	French, Italian, Polish, Swiss and British national runners (851,647 season-best performances)	Speed; CS, * D*′	CS: 0.83; < 0.001 *D*′: 0.02; < 0.001	
1000 m	Kolbe et al. [16]	17 male distance runners; 31.7 ± 7.3 years old, $\dot{V}$O_2max_ 59.2 ± 4.6 mL/kg/min	Time: CS	− 0.75; < 0.001	
1500 m	Bellinger et al. [134]	21 male, 11 female trained middle distance runners; $\dot{V}$O_2peak_ 72.1 ± 3.2 mL/kg/min, $\dot{V}$O_2peak_ 61.2 ± 3.7 mL/kg/min	Overall time and last lap performance during fast, moderate, and slow 1500 m trials. Males and females: CS and * D*′	Refer to the original study for all relationships. Only significant findings are reported here Overall time in the fast 1500 m CS: 0.69, < 0.05 (males); 0.86, < 0.05 (females) Last lap performance in the fast 1500 m CS: 0.55, < 0.05 (males); 0.66, < 0.05 (females)	There were mixed findings for the association between CS and 1500 m time. A faster CS was correlated to a faster 1500 m time in 3 of 4 studies, in addition to last lap time, but not overall time in moderate and slow trials *D*′ was not deterministic of overall or last lap time in fast, moderate, or slow trials (*n* = 1); weakly associated with speed (*n* = 1)
	Dearing and Paton [91]	16 males, 4 females. Two internationally competitive, 3 national class, 15 recreational runners; 39.5 ± 14.6 years old, $\dot{V}$O_2peak_ 3.93 ± 0.73 L/min	Time: CP	− 0.93; < 0.01	
	Pompeu Trevelin et al. [133]	20 male university athletes; 22.9 ± 3.0 years old, $\dot{V}$O_2max_ 46.2 ± 4.9 mL/kg/min	Time: CS	Only significant correlations reported *p* > 0.05	
	Walt et al. [131]	French, Italian, Polish, Swiss and British national runners (851,647 season-best performances)	Speed; CS, * D*′	CS: 0.89; < 0.001 *D*′: 0.04; < 0.001	
1600 m	Pettitt et al. [49]	14 collegiate female distance runners; 19 ± 1 years old, $\dot{V}$O_2max_ 55 ± 4 mL/kg/min	Race speed above CS: * D*′	0.79; NR	
3000 m	Guimaraes and Da Silva [109]	19 male runners; 29.3 ± 3.2 years old	Time: CS	− 0.94; < 0.05	A higher CS was associated with a faster 3.000 m speed (*r* = 0.97–0.99 in all 4 studies) and improved time (*n* = 1) *D*′ was weakly and negatively associated with speed, although limited to 1 study
	Massini et al. [66]	7 athletes; 15.3 ± 1.4 years old, $\dot{V}$O_2max_ 54.2 ± 5.2 mL/kg/min	Speed: CS	0.99; 0.001 (R^2^ adjusted)	
	Silva et al. [110]	11 physical education students; 20.7 ± 1.8 years old, $\dot{V}$O_2max_ 48.9 ± 5.8 mL/kg/min	Speed: CS	0.99; < 0.0001	
	Simões et al. [92]	20 male endurance runners; 20.7 ± 1.8 years old	Speed: CS	0.99; < 0.0001	
	Walt et al. [131]	French, Italian, Polish, Swiss and British national runners (851,647 season-best performances)	Speed; CS, * D*′	CS: 0.97; < 0.001 *D*′: − 0.15; < 0.001	
5000 m	Dearing and Paton [91]	16 males, 4 females. Two internationally competitive, 3 national class, 15 recreational runners; 39.5 ± 14.6 years old, $\dot{V}$O_2peak_ 3.93 ± 0.73 L/min	Time: CP	− 0.94; < 0.01	An increased CS was associated with a reduced performance time (*r* = − 0.90 to − 0.98, * n* = 5), in addition to and increased speed (*r* = 0.82–0.98, * n* = 2). However, in a race context, CS itself may not be deciding factor on race position (*n* = 1) *D*′ was negatively associated with, or not correlated with 5000 m speed (*n* = 2), or race finishing position (*n* = 1), but was associated with the speed held above CS (*n* = 1). * D*′ balance throughout a race may be more indicative of finishing position (*n* = 1)
	Figueiredo et al. [95]	25 male recreational runners; 28.6 ± 4.7 years old	Time: CS (derived from multiple trials; CS modelled from the 1000, 1800, 2600 m trials reported)	− 0.95; * p* < 0.05	
	Follador et al. [135]	36 recreational endurance runners; 32.2 ± 6.2 years old; $\dot{V}$O_2max_ 53.3 ± 6.1 mL/kg/min	Time: CS	− 0.90; < 0.001	
	Guimaraes and Da Silva [109]	19 male runners; 29.3 ± 3.2 years old	Time: CS	− 0.97; < 0.05	
	Kirby et al. [5]	2017 IAAF World Championships 5000 m men’s finalists	Finishing position: CS; * D*′; * D*′ balance	CS: 0.2; 0.58 *D*′: > 0.05 *D*′ balance: from 400 m onwards ~ 0.7 and above, < 0.05 from 400 m onwards *D*′ balance at final 400 m: 0.97; < 0.001	
	Nimmerichter et al. [34]	16 male trained endurance athletes; 30.4 ± 7.3 years old; $\dot{V}$O_2peak_ 4.8 ± 0.6 L/min	Speed: CS; * D*′	CS: 0.82; < 0.01 *D*′: − 0.002; > 0.05	
	Pettitt et al. [49]	14 collegiate female distance runners; 19 ± 1 years old, $\dot{V}$O_2max_ 55 ± 4 mL/kg/min	Race speed above CS: * D*′	0.97; NR	
	Pompeu Trevelin et al. [133]	20 male university athletes; 22.9 ± 3.0 years old, $\dot{V}$O_2max_ 46.2 ± 4.9 mL/kg/min	Time: CS	− 0.98; < 0.05	
	Walt et al. [131]	French, Italian, Polish, Swiss and British national runners (851,647 season-best performances)	Speed; CS, * D*′	CS: 0.98; < 0.001 *D*′: − 0.35; < 0.001	
10,000 m	Corrêa et al. [136]	20 male, 14 female runners; 42.4 ± 11.0 years old	Speed: CS	0.93; NR	A higher CS was consistently associated with lower performance time (*r* = − 0.85 to − 0.95, * n* = 3), greater trial speed (*r* = 0.8 to 0.99, *n* = 5), and race finishing position (*r* = 0.62 to 0.82, * n* = 2) A larger * D*′ is moderately associated with a reduced speed (*n* = 1). * D*′ does not seem to be deterministic of race finish position (*n* = 1). A larger within-race * D*′ balance was associated with race finishing speed (*n* = 1) and finishing position (*n* = 1)
	Follador et al. [135]	36 recreational endurance runners; 32.2 ± 6.2 years old; $\dot{V}$O_2max_ 53.3 ± 6.1 mL/kg/min	Time: CS	− 0.95; < 0.001	
	Foster et al. [137]	35 finishers of the 2008 Men’s Olympic 10,000 m	Finishing position, mean speed, and finishing speed over last 1000 m: CS*; * D*′ balance*	Finishing position CS: * r* = 0.82; NR Mean speed CS: *r* = 0.80; NR Finishing speed over the last 1000 m *D*′ balance: *r* = 0.87; < 0.05	
	Kirby et al. [5]	2017 IAAF World Championships 10,000 m men’s finalists	Finishing position: CS; * D*′; * D*′ balance	Finishing position CS: 0.62; < 0.05 *D*′: > 0.05 *D*′ balance: from 2800 m onwards 0.55; < 0.05 Final 400 m * D*′ balance and finishing position: 0.97; < 0.001	
	Kolbe et al. [16]	17 male distance runners; 31.7 ± 7.3 years old; $\dot{V}$O_2max_ 59.2 ± 4.6 mL/kg/min	Time: CS	− 0.85; < 0.00001	
	Kranenburg and Smith [99]	9 highly trained male runners; 26 ± 5 years old; $\dot{V}$O_2max_ 67.7 ± 4.1 mL/kg/min	Speed: CS (track, and inverse-time model derived CS)	0.92; 0.0005	
	Santos et al. [138]	9 males, 3 female runners; 36.0 ± 4.8 years old; $\dot{V}$O_2max_ 60.1 ± 7.8 mL/kg/min	Time: CS	− 0.86; < 0.001	
	Simões et al. [92]	20 male endurance runners; 20.7 ± 1.8 years old	Speed: CS	0.902; < 0.0001	
	Walt et al. [131]	French, Italian, Polish, Swiss and British national runners (851,647 season-best performances)	Speed; CS, * D*′	CS: 0.99; < 0.001 *D*′: − 0.48; < 0.001	
Half marathon	Kolbe et al. [16]	17 male distance runners; 31.7 ± 7.3 years old; $\dot{V}$O_2max_ 59.2 ± 4.6 mL/kg/min	Time: CS	− 0.79; < 0.001	A higher CS was associated with a reduced performance time (*r* = − 0.79 to − 0.93, * n* = 2), and greater running power (*n* = 1)
	Olaya-Cuartero et al. [102]	9 trained athletes; 38.1 ± 5.4 years old	Power: CP	0.88; 0.002	
	Santos et al. [138]	9 males, 3 female runners; 36.0 ± 4.8 years old; $\dot{V}$O_2max_ 60.1 ± 7.8 mL/kg/min	Time: CS	− 0.93; < 0.001	
Marathon	Florence and Weir [139]	6 male, 6 female runners; 29.2 ± 3.9 years old	Time: CS	− 0.87; NR	A greater CS was associated with a faster performance time (*n* = 2)
	Smyth and Muniz-Pumares [53]	31,190 runners from 2014–2017 Dublin, London, and New York marathon runners	Finish time and relative marathon speed (marathon speed/ CS)	− 0.99; < 0.01	

*CP* critical power, *CS* critical speed, *IAAF* International Association of Athletics Federations, *NR* not reported, $\dot{V}$*O*_*2max*_ maximal oxygen consumption, $\dot{V}$*O*_*2peak*_ peak oxygen consumption

### Application of CS and *D*′ in Training

Fifteen studies investigated the application of CS or *D*′ concepts in a training context. Current applications include prescribing exercise intensity based on CS or using CS as an intensity landmark (i.e., intervals performed above and below CS during work and recovery bouts, respectively; *n* = 13) [3, 7, 80, 87, 88, 122–129]; prescribing work bouts as a function of *D*′ depletion (*n* = 3) [123, 128, 130]; and prescribing work bouts as a fraction of TTE via modelling (*n* = 3) [23, 87, 126]. Of these interventions, ten were applied in an acute setting [3, 7, 23, 80, 87, 88, 122, 124, 127, 129], and five applied CS and *D*′ concepts over a training intervention [123, 125, 126, 128, 130].

## Discussion

The aims of this scoping review were to determine: (1) the specific testing protocols and modelling methods that have been implemented to quantify CS and *D*′ in running; (2) the predictive potential of CS and *D*′ for running race performance; and (3) the application of CS and *D*′ in individualising running training prescription. Fundamentally, CS has been validated as the boundary between the heavy and severe domains, and, therefore, can differentiate steady-state and non-steady-state exercise intensities during running. Furthermore, there is a large body of research investigating the measurement and modelling of CS and *D*′; however, the testing protocols varied widely, and included differences in the task types used (time trials (TTs), time to exhaustion tasks (TTE), 3MT, and T10), the number and length of exercise trials used, testing surfaces, and subsequent modelling methods. Comparatively, there is a far smaller body of research investigating *D*′ reconstitution. The influence of CS and *D*′ on determining performance has spanned from the 100 m to the marathon. CS appears to be a particularly strong predictor of performance (for distances ≥ 1000 m), although initial insights suggest *D*′ balance modelling may be more deterministic of race finish position (investigated during the 5000 and 10,000 m), and provides additional physiological context to pacing and tactics. CS and *D*′ prescription concepts (i.e., prescribing intensity relative to CS, or modelling *D*′ depletion or TTE) have been used to assess the acute and chronic physiological response of training interventions, and the implementation of modelling to assist in individualised training prescription.

### CS and *D*′ Measurement: Validating Critical Speed as the Maximal Metabolic Steady State

There is consistent evidence supporting CS as the boundary between the heavy- and severe-intensity exercise domains, often inferred from the $\dot{V}$O_2_ [30, 73, 90, 96, 106, 118] or blood lactate [98, 106] responses to exercise performed at sub- or supra-CS intensities. Given that CS is intended to represent the boundary between the heavy- and severe-intensity exercise domains (i.e., differentiate steady-state and non-steady-state exercise intensities), comparisons have been made to previously established tests and physiological landmarks (Table 1). However, there were often discrepancies between CS and other physiological thresholds, which is consistent with the cycling literature [11, 140]. Although CS and other physiological thresholds are inter-related, variations in the intensity at which they occur may also arise from different underlying physiological mechanisms. For example, the RCP represents the point at which an increase in metabolic acidosis drives a compensatory hyperventilation [141], but does not necessarily distinguish steady- versus non-steady-state physiology. For this same reason, threshold equivalence does not necessarily validate the specific parameters as the heavy-severe boundary.

Furthermore, differences between cycling CP and other constructs thought to represent the MMSS may be due to differences and inconsistencies in testing methodology [140]. Indeed, several studies comparing CS to other measures of the MMSS may have employed trials that were too short (i.e., the shorter trials were less than 2 min, and longer trials were less than 12 min) [103, 105, 110–112], thus resulting in an inflated CS (as opposed to 3- and 15-min trials; see ‘Task Duration’ discussion in Sect. 4.1.1). Therefore, differences between CS and physiological thresholds may have arisen due to variations in the protocols implemented to estimate CS. Within a practical setting for the aforementioned reasons, CS and other physiological thresholds are not interchangeable. Therefore, the same physiological parameter should be used when assessing physiological changes over time. Additionally, pre-set prescriptions at a percentage of CS and LT2, for example, cannot be used synonymously. This review provides a contemporary resource to inform practitioners about the relatability and comparison between different physiological testing methods for the MMSS in running.

#### Factors Impacting CS and D′ Measurement

*Effect of the model:* The comparison of the validity of model estimates has been typically inferred from or suggested via standard error, metabolic responses (i.e., $\dot{V}$O_2_ consumption), and comparisons of modelled to actual performance. The three-parameter hyperbolic and the exponential models have demonstrated larger standard errors compared to the two-parameter models [29, 31, 35, 90]. No studies have directly compared two-parameter and three-parameter sub- and supra-CS $\dot{V}$O_2_ consumption (to validate CS estimates as the heavy-severe exercise intensity transition). However, CS derived from two-parameter models has been repeatedly verified as the MMSS (based on $\dot{V}$O_2_ consumption) [30, 73, 106, 118]. The evidence supporting a specific model based on the predictive accuracy is mixed [13, 23, 24, 38, 43], with all models showing promising results at times, although two-parameter models tend to provide similar performance predictions between each other [24, 25, 28, 34, 43]. Furthermore, the predictive accuracy of all models may be dependent upon the length of trials used to calculate CS and *D*′ estimates [38]. The three-parameter hyperbolic and exponential model has a stronger association with 800 m performance compared to the two-parameter models [24]. However, the 800 m is performed outside the typical range for optimal race estimation using CS modelling (i.e., above the severe intensity domain). Therefore, the addition of maximal sprint speed may have improved the 800 m performance estimation given the strong relationship between maximal sprint speed and performance in the extreme intensity domain [142].

There is consistent evidence suggesting that CS and *D*′ values derived from the linear distance-time, speed inverse-time, and two-parameter hyperbolic models are comparable [13–15, 21, 24, 27–31, 35–37, 40], with a relatively smaller conflicting evidence base [22, 25, 26, 34, 39]. The three-parameter hyperbolic model tends to overestimate *D*′ (in relation to a statistically or practically meaningful difference to the two-parameter models), often coinciding with a smaller CS [13, 23, 24, 29, 31, 33, 35, 37, 40]. In support of this difference between models, the TTE at CS derived from the three-parameter model has been shown to be approximately 52 min [90], which would correspond to exercise performed in the heavy-exercise intensity domain. An inflated *W*′, and lower CP from the three-parameter model compared to two-parameter models is also evident within the cycling literature [143, 144]. The exponential model has shown inconsistency for CS measurement compared to the two-parameter models [24, 29, 31, 35] (i.e., CS from the exponential model was faster (*n* = 2), the same, or slower than two-parameter model estimates). *D*′ tends to show larger between-model variance compared to CS. This is likely due to the modelling parameter associated with *D*′ (i.e., the curvature constant or y-intercept) being particularly sensitive to the variability of the input data. Therefore, trials should be performed under as similar conditions as possible to reduce variability between trials.

Given that two-parameter models provide a more valid estimate of CS and *D*′ than the three-parameter hyperbolic model, and can be used to accurately predict performance, their use is recommended. Although CS, *D*′, and performance estimates tend to be similar between the two-parameter models, there is support for using the inverse-time model [13]. Ruiz-Alias et al. [13] compared the work performed above running CP (i.e., *W*′) during an actual 4 min TT performance to the estimated TTE associated with 4 min TT work rate (i.e., running power) derived from the distance- and inverse-time models, for the validation of the *W*′ estimate from each model [13]. Both models closely estimated 4 min TTE work rate; however, the inverse-time model, in particular, showed the least error for work performed above CP during the 4 min TT, suggesting a more accurate estimate of *W*′. However, this study investigated CP and *W*′; whether these results are consistent when modelling CS and *D*′ remains unknown. Furthermore, the work performed above CP or CS, and the associated error, may vary with task duration [50]. As such, further research is required to assess if *D*′ is indeed a variable and not a constant, or if alterations in *D*′ across the speed-duration curve are in fact due to poor validity of measurement.

*3MT versus multi-trial testing:* The 3MT is useful for determining CS, *D*′ and maximal sprint speed from a single test. CS derived from the standard 3MT is generally consistent with multi-trial testing procedures, although *D*′ tends to be underestimated by the 3MT [26, 27, 42, 60, 73–75]. Additionally, performing the 3MT with shuttles results in a meaningfully different CS and *D*′ estimate, compared to the traditional 3MT [62–64, 84]. Prescribing supra-CS exercise based on these estimates should be approached with caution considering *D*′ is under-estimated.

*Task duration:* The duration of tasks performed impacts CS and *D*′ estimates. Alterations in trial duration, and resulting CS (and *D*′), have been reported in 12 studies [18, 28, 38, 41, 54, 55, 58, 66, 68, 70, 95, 97]. CS is sensitive to the length of the longest trial used within the model, whereby an increased duration of the longest trial will reduce CS [68, 70, 97]. Additionally, it has been proposed that CS may correspond to 95–99% of the speed of the longest trial length used [97]. Given that CS demarcates the heavy- and severe-exercise intensity domains, and that CS is ~ 95% of the longest trial used, all trials should be completed at speeds faster than CS (i.e., in the severe domain). Furthermore, the *D*′ estimate will alter depending on the duration of the trials, in which a smaller CS (i.e., longer duration trial used) will coincide with a larger *D*′, and vice versa. Given that altering the durations of the trials used will affect the estimation of CS and *D*′, a consistent protocol is required for repeat testing of athletes. It is recommended that testing protocols include: (1) at least three maximal effort trials of different durations performed in the severe domain to obtain an error estimate, (2) a shorter effort trial approximately 3 min in duration, in which $\dot{V}$O_2max_ is obtained, and (3) a longer effort approximately 15 min in duration as the TTE at CS is approximately 16.5–20 min [47, 106], and therefore, exercise should be of a shorter duration to remain in the severe domain. These recommendations should be extended to CS and *D*′ estimates modelled from elite athlete-race data, whereby events ≤ 800 m and ≥ 10 km should not be included as a predictive trial. Using standardised fixed duration trials, as opposed to distance-based trials, would minimise alterations in task length having an impact on the modelled CS and *D*′, such as for evaluating within-athlete fitness changes, and between-athlete fitness differences. However, distance-based trials may be more reliable (due to familiarity) [145], and are easier to implement and obtain data from a practical point of view (i.e., using competitive race performances).

*Task surface and task type:* Five studies investigated the difference between surface type (i.e., track or treadmill specifically) [58, 66, 67, 71, 99], and six have investigated the impact of task type (i.e., TT, TTE, or training/game data) [58, 60, 66, 71, 99, 100] on CS and *D*′ measurement. Comparisons between the effect of track or treadmill testing, and TT or TTE tests were often completed in tandem, with limitations of study designs (e.g., various test lengths used, and altering both the surface and task type simultaneously) making conclusions difficult.

Over-ground (i.e., track) and treadmill-derived CS and *D*′ estimates from trials with similar duration were investigated in two studies [67, 71]. However, there is conflicting evidence on the transferability between track and treadmill-derived CS and *D*′. Ruiz-Alias et al. [67] reported that running CP increased, but *W*′ decreased, from track TTs compared to treadmill TTs. In contrast, Triska et al. [71] reported no significant differences in CS or *D*′ between running modality (i.e., over-ground track vs. treadmill). Regarding TT versus TTE studies, only one study used same-duration testing, which provided similar CS and *D*′ estimates [71]. Other studies comparing TT and TTE tasks found that while CS remained similar [99], *D*′ was larger when derived from the TTE tasks [58], or that there was a practically meaningful difference in CS and *D*′ [66] (higher CS and lower *D*′ from treadmill testing – although not statistically different; possibly due to differences in trial lengths used between conditions). Furthermore, using GPS-derived match data (soccer) [100] provides a similar CS estimate to TT-derived CS. Additionally, CS and *D*′ that were estimated from habitual running training data [60] are comparable to estimates from TT. As such, training and match data may serve as non-invasive, or ‘invisible monitoring’, testing strategies to obtain CS and *D*′ estimates. Given that current findings surrounding task surface and type are largely contextual, testing procedures should ideally be performed on the same modality/surface as training or races to ensure confidence and transferability of the estimated parameters to the applied setting or performance modelling. This is further supported by athletes achieving greater task reliability when the task type is familiar [145] (i.e., using set-distance time trials that the athlete has previously performed).

*Inter-task recovery and trial order:* Performing multiple exhaustive trials causes fatigue and can be disruptive to training schedules. As such, the validity of single-day, multi-trial testing has been investigated. Initial findings suggest that same-day testing has no impact on running CP or *W*′ estimation compared to multi-day testing (3- and 9-min TTs, interspersed by 30 min of recovery) [67]. Galbraith et al. [58] reported that single-day testing resulted in a compromised *D*′ measurement; however, the testing compared track TT to treadmill TTE tasks of different durations such that differences in the testing protocols may have influenced the resulting CS and *D*′ estimates. Nevertheless, the study also reported that there was no difference between 30 or 60 min of recovery between multiple TTs performed on the same day for determining CS and *D*′. Given the findings of Ruiz-Alias et al. [67] and Galbraith et al. [58], 30 min seems a sufficient recovery period between multi-trial testing; however, the difference between CS and *D*′ estimates from same-day and multi-day testing when there are three or more trials (under the same conditions, i.e., surface and task type) needs clarification.

Following on, the impact of same-day trial order has been minimally investigated. Performing a 3-min TT has been shown to impair a subsequent 9-min TT, whereas performing the 9-min TT first had no impact on the 3-min TT [67]. Therefore, a greater running power was obtained when the 9-min TT was performed first, increasing the running CP estimate and reducing *W*′. Other investigations have tended to order the trials from longest to shortest duration [58, 70, 71], but have not specifically investigated the trial order. Future research may explore the impact of trial order when three or more TTs are used, which allows for the attainment of error estimation.

*Impact of external variables:* Additional factors impacting CS and *D*′ measurement have also been investigated. High environmental heat reduces CS without impacting *D*′ [65], and the 3MT is reliable in hot conditions [84]. Increased surface gradient [57], and load carriage [69], also reduce CS without impacting *D*′.

#### *D*′ Reconstitution

There is currently a narrow literature base concerning *D*′ reconstitution and *D*′ balance modelling in running (six studies [3, 7, 80, 87–89]), particularly when compared to the work completed in cycling [10]. Both linear [7, 80], and exponential models [3, 7, 87–89] of *D*′ reconstitution have been examined, with strong evidence suggesting *D*′ reconstitution follows an exponential course of recovery [3, 88]. There are mixed results between the models used to estimate TTE and *D*′ balance, although the *W*′ balance model proposed by Skiba et al. [2] shows promising results when a running specific recovery time constant is applied to the model [3, 7, 87, 89].

The *D*′ balance model has been expanded by deriving a recovery-intensity-specific time constant [3, 7], given that the proximity of the recovery intensity to CS alters the reconstitution rate of *D*′ [3, 89]. However, these investigations were based on running power [3], or did not assess the recovery time constant through different intensity domains [7]. A time constant based on running speed in different intensity domains may be needed, given that running speed is more widely used by coaches and athletes. Additionally, there was a large variation between the individual reconstitution responses and the modelled group time constant [3]. Given that the participants exhibited a wide range in fitness ($\dot{V}$O_2max_ range of 16 ml/kg/min), and that athlete fitness and performance calibre can dictate reconstitution kinetics [146, 147], time constants for athletes with distinctly similar physiological characteristics or running performance need to be determined.

Time constants for *W*′ reconstitution in cycling have been reported as 377, 452 and 578 s for recovery at 20 W and the moderate and heavy exercise intensity domains, respectively [2]. The relationship between the time constant and difference between recovery power output and CP is denoted by *τ* = 546e^(−0.01DCP)^ + 316 [2]. In comparison, running recovery at light intensities, and in the moderate and heavy intensity domain, yield time constants of 119, 190 and 336 s, respectively, which can be described through the equation *τ* = 372e^(−0.02DCP)^ + 102 [3]. Further running-specific time constants are listed in Table 5. These findings suggest that *W*′ reconstitution occurs at a faster rate in running than cycling, although this notion is in contrast to the findings of Bellenger et al. [89]. Although the influence of work-bout duration [88] and recovery intensity [3] have been investigated, the effect of *D*′ depletion rate during the work bout on subsequent reconstitution also requires attention, since a faster *W*′ depletion rate has been shown to increase *W*′ reconstitution rate in cycling [148]. Additionally, the intensity-duration relationship can alter depending on prior work and fatigue [149], and accounting for such changes can improve the accuracy of *W*′ modelling [150]. The influence of dynamic changes in CS, *D*′ and recovery kinetics (i.e., the recovery time constant) due to fatigue within a running session on *D*′ balance modelling warrants investigation. Furthermore, all running-based studies have utilised a mono-exponential reconstitution of *D*′, but Chorley et al. [151] suggest *W*′ reconstitution in cycling may be better characterised by a bi-exponential model.

Based on current evidence, both the Skiba et al. [2] integral or differential equation can be used to quantify *D*′ balance and proximity to exhaustion during intermittent running [3, 89]. Practitioners employing the modelling will currently need to quantify an individual-specific, running-based recovery time constant which can be scaled to recovery intensity below CS. Additionally, current modelling may not be accurate during short, higher-intensity intervals (i.e., > 150% CS) [7], although inaccuracies in quantifying *D*′ balance in such interval sessions may be due to the interval intensity falling outside of optimal CS modelling ranges (i.e., above the severe intensity domain). Furthermore, as variability is still present between exhaustion coinciding with a *D*′ balance of 0 m, a threshold for predicting exhaustion of 25 m (~ 10% of *D*′) has been suggested [89]. The error in current modelling also needs to be considered in race-strategy simulation, whereby a conservative approach may be taken to ensure the athlete does not finish a race with a modelled *D*′ balance of 0 m, as this may eventuate in premature exhaustion.

#### Intermittent CS Versus CS

The intermittent CS (iCS) is “the velocity that a person can sustain during repeated exercise bouts without eliciting fatigue” [78]. The iCS concept may provide a framework for practitioners to accurately predict time to exhaustion during intermittent running, especially given current limitations around *D*′ reconstitution modelling. Testing protocols typically involve standardised work:rest intervals performed to exhaustion (e.g., 30:15 s) at a given work-bout intensity, with the intensity of the work-bout changing each trial (e.g. 110, 120, 130% maximal aerobic speed). The distance-time model is typically used to describe the relationship between the distance covered and time to exhaustion in each trial, and thus derive iCS (slope) and intermittent *D*′ (i*D*′; *y*-intercept) The estimated iCS and i*D*′ can subsequently be used to model proximity to exhaustion within an interval training session. Nine studies have investigated the iCS and intermittent *D*′ (i*D*′) [26, 56, 76–80, 98, 152].

However, iCS and i*D*′ are not physiologically equivalent to CS and *D*′. iCS has been shown to be greater than (*n* = 1 [76]), equal to (*n* = 1 [79]), or lower than CS (*n* = 2 [80, 98]). i*D*′ has been shown to be higher than *D*′ during iCS tests (*n* = 3 [76, 79, 80]). The variability in results is likely due to the array of intermittent testing protocols, which can influence parameter outcomes [56]. As such, if the iCS is used for training prescription, the testing protocol should remain consistent with the desired training session (e.g., 15:15 s for team sport athletes), so that the obtained estimates are applicable to the session.

### CS and *D*′ as a Determinants of Running Performance

CS and *D*′ are determinants of performance across a range of distances (400 m-marathon). Although simple correlations provide insight as to whether pre-race CS and *D*′ impact overall race time, further exploration of pacing at various stages throughout a race may provide additional insight as to how physiological characteristics can impact performance. Bellinger et al. [132, 134] investigated the impact of CS and *D*′ on overall and last lap time during fast, moderate and slow 1500 m TTs, and during fast and paced 800 m TTs. There was no correlation between CS or *D*′ and overall or last lap performance, except for CS and overall finishing time in the fast 1500 m trial. These nuanced conditions (i.e., alteration in race pace and tactics) highlight the complexity of predicting race performance.

This association between *D*′ and 800 and 1500 m performance suggests that the overall size of *D*′ at the beginning of a race may not necessarily be a predictor of finishing time. However, the amount of *D*′ used and the amount remaining (*D*′ balance) in competitive races may further explain the individual physiological demand and be a better determinant of finishing position. Kirby et al. [5] reported a strong correlation between within-race *D*′ balance and 5000 and 10,000 m running performance (albeit with no correlation between pre-race *D*′ magnitude and finishing position). Investigations involving CS, *D*′ and *D*′ balance and race performance should be expanded across other distances, and to various racing tactics, to determine the influence of these variables under given race contexts. These physiological parameters can subsequently be used to inform race strategy and pacing to take advantage of an athlete’s physiological characteristics. The use of CS and *D*′ to inform race strategy is also discussed by Pettitt [153].

### The Application of CS and *D*′ to Individualise Training

CS and *D*′ concepts can provide an additional avenue to individualise training prescription. Current applications of CS and *D*′ in training have largely centred around prescribing running relative to CS. The use of CS as a landmark or prescribing exercise as a percentage of CS allows for intensity to be prescribed as sustainable or steady state (< CS), or non-sustainable (> CS). Additionally, studies have prescribed exercise intervals based on a given *D*′ depletion or TTE, adding a further layer of individualisation [23, 87, 123, 126, 128, 130]. As such, intervals may be prescribed as a fixed % of CS, a fixed fraction of a TTE of a given speed, or via *D*′ balance modelling (see Table 7).

**Table 7:** Example interval set designs for a middle- or long-distance runner

Prescription method	Example set design
Fixed % of CS	6 × 1 km @ 105% CS. 60 s passive recovery [154]
Fixed fraction of TTE	6 × 60% TTE of 115% CS. 2:1 work: rest [155]
*D*′ balance modelling	3, 2, 1, 1, 1, 1 min etc. @115% CS. 2:1 work:rest until 25% * D*′ balance (i.e., hard but not exhaustive) [88]

*CS* critical speed, *TTE* time to exhaustion

Intervals performed at a fixed % of CS do not consider how well an individual athlete can sustain the given intensity and therefore, require the expertise of a coach or previous experience of the athlete to manipulate the set design to achieve the desired response. For example, two athletes with the same CS but different magnitudes of *D*′, running at the same speed above CS will incur a different proximity to task failure and physiological cost (i.e., intramuscular metabolite accumulation [156, 157]) which needs to be considered.

Understanding and modelling the TTE for a given intensity may help to prescribe training, considering the known literature. For example, the time to achieve $\dot{V}$O_2max_ is approximately 50% TTE for the given severe intensity work bout [23]. Given that maximising time spent at high fractions of $\dot{V}$O_2max_ may be an important stimulus for its improvement [158], modelling can be used to accrue time at/above 50% TTE (i.e., 0–50% *D*′ balance). Additionally, performing intervals to 60% TTE of v$\dot{V}$O_2max_ can provide superior adaptations (i.e., greater improvements in the ventilatory threshold, and TTE at v$\dot{V}$O_2max_) and performance improvements (i.e., greater improvements in 3000 m) than intervals performed to 70% TTE [159]. However, performing intervals at a given fraction of TTE does not consider the recovery between intervals and therefore, cannot ascertain proximity to exhaustion past the first interval (depending on the fixed fraction of TTE chosen). *D*′ balance modelling provides the most individualised interval prescription. Proximity to exhaustion can be estimated at any given point during the session, and the impact of work and recovery characteristics can be modelled prior to the session to assess if the prescription aligns with the desired session outcome. However, *D*′ balance modelling is the most labour-intensive method prior to a training session and requires the calculation of an individualised recovery time constant. As such, practitioners may wish to consider such information to inform their training prescription. Similar applications of CS methods have been explored by de Aguiar et al. [124] and Vaccari et al. [88], whereby the relationship between exercise intensity and duration have been exploited to increase the time spent at high fractions of $\dot{V}$O_2max_ during intermittent running sessions. Further discussion on the use of CS and *D*′ for prescribing HIIT has also been summarised previously [153, 160].

**Fig. 2:**
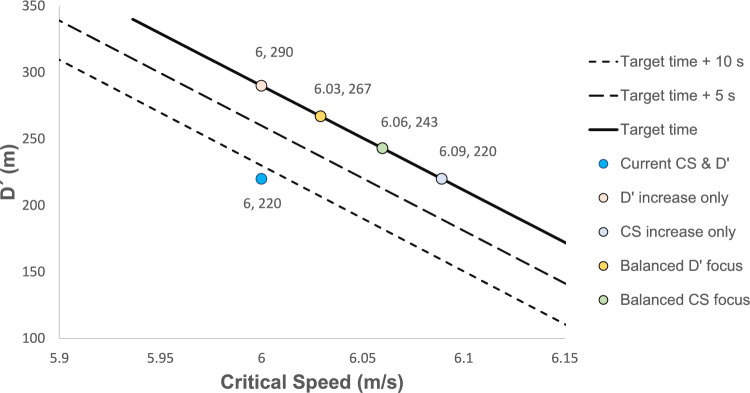
The current CS and *D*′ of a hypothetical athlete (light blue marker) and the required changes needed in CS and/or *D*′ to achieve a goal performance time of 13:05 for 5000 m (solid black line). *CS* critical speed

Furthermore, the modelling of the required changes to CS and *D*′ to achieve a target race performance can be used to prospectively plan training. For example, CS and *D*′ modelling can inform how much of an increase in CS, *D*′ or both would be required to meet qualifying standards, or achieve a podium-worthy performance time. Figure 2 provides an example of a hypothetical athlete aiming to meet 5000 m qualifying standards for the Paris 2024 Olympic Games with a time of 13:05. From there, training can be tailored to target the specific improvements in CS or *D*′. Additionally, training monitoring approaches can be utilised retrospectively to determine how previous interventions have influenced changes to CS and *D*′ within a season.

Five studies investigated the effect of a chronic training intervention, typically showing improvements in CS but not *D*′ [123, 125, 126, 128, 130]. Training prescription of repetitions performed for a duration of 60–90 s and at speeds above 140% CS has been suggested as a possible avenue to improve *D*′ [128], but has not been directly assessed. A deeper understanding of *D*′ determinants would assist in informing training interventions to target *D*′ improvement. Interestingly, no studies have applied *D*′ balance models within training longitudinally. However, this lack of representation may be due, in part, to the limitations of current methods for quantifying interval session *D*′ balance. Quantifying training stimulus through sessional *D*′ balance over a chronic training period or season may provide insight into optimising training for performance and physiological adaptations.

### Limitations

Given that scoping reviews are intended to summarise a field of literature, rather than quantitatively answer a specific question (such as a meta-analysis), a statistical synthesis of the difference between CS and other physiological thresholds was not undertaken. However, this review highlights that there currently exists a discrepancy between CS and other measurements of physiological thresholds. This review provides a resource for practitioners to locate and assess the current evidence on this topic, while also providing a summary. A further limitation to this study, and scoping reviews in general, is the limited focus on quality assessment of the included studies, such as a risk of bias appraisal. As such, studies within this review may have been used to draw comparisons with related literature without considering the methodological quality of the study. Additionally, non-English studies and grey literature were not included in this review, which may result in selection bias. This may limit the findings particularly in cases where the evidence base is small, such as *D*′ reconstitution.

Furthermore, there are under-represented groups across the literature such as females, Masters, and para-athletes. Additionally, the impact of heat and altitude on the acute responses, and time-course adaptations to CS and *D*′ have not been investigated. The effect of advanced footwear technology (i.e., ‘super shoes’) also requires additional attention. A previous study investigating advanced footwear technology included a half-marathon as a predictive trial and, therefore, did not adhere to recommended modelling protocols. Future research may compare differences between shoes designed for shorter races (i.e., mile to 10 km) with longer races (half-marathon and marathon), and non-race-specific shoes on CS and *D*′ estimates in conjunction with recommended modelling protocols.

Furthermore, although studies that included a model-predicted and actual performance time comparison were eligible for this review, only studies that compared performance prediction from multiple models were discussed in the article. Studies investigating a single model can provide insight as to the capability of the model for predicting performance (and providing valid CS and *D*′ estimates), but not necessarily if it is the optimal model. Although not discussed, these studies assist in determining the validity of the model used.

## Conclusion

This review has summarised the measurement methods, influence on performance, and application of CS and *D*′ in running. CS accurately identifies the heavy-severe intensity domain transition. The most valid estimates of CS and *D*′ are typically derived with two-parameter models using predictive trials with bounds of approximately 3 and 15 min. However, the assessment of *D*′ variability depending on task duration requires further attention. The Skiba et al. [2] integral and differential equations [3, 89] can be used to quantify intermittent running *D*′ balance when a running-specific time constant is applied. However, additional work is required to improve discrepancies between *D*′ balance modelling and task exhaustion. Potential contextual factors impacting *D*′ reconstitution, such as *D*′ depletion rate, need clarification. *D*′ balance appears promising for physiologically contextualising and predicting race performance (5000 and 10,000 m), but research needs to be completed across different running distances (e.g., middle-distance events) and race tactics to assess the deterministic potential of *D*′ balance on race performance in various contexts. CS and *D*′ modelling provides an avenue to individualise training prescription and a framework to assess and target performance time improvement and physiological adaptations. However, no literature has assessed training interventions where *D*′ balance modelling has been implemented longitudinally.

## Supplementary Information

Below is the link to the electronic supplementary material.

Supplementary file1 (DOCX 47 KB)

## Declarations

### Availability of Data and Material

Data are available from the authors upon reasonable request. The data extraction sheet is available from the OSF registry. No data or code were used for figure production and analysis.
